# Prevalence and predictors of stroke among individuals with prediabetes and diabetes in Florida

**DOI:** 10.1186/s12889-022-12666-3

**Published:** 2022-02-06

**Authors:** Md Marufuzzaman Khan, Shamarial Roberson, Keshia Reid, Melissa Jordan, Agricola Odoi

**Affiliations:** 1 Department of Public Health, College of Education, Health, and Human Sciences, University of Tennessee, Knoxville, TN USA; 2 Florida Department of Health, Tallahassee, FL USA; 3 Department of Biomedical and Diagnostic Sciences, College of Veterinary Medicine, University of Tennessee, Knoxville, TN USA

**Keywords:** Prediabetes, Diabetes, Stroke, Predictors, Risk factors, Logistic regression, Behavioral Risk Factor Surveillance System, BRFSS, Florida, United States, USA

## Abstract

**Background:**

The prevalence of both prediabetes and diabetes have been increasing in Florida. These increasing trends will likely result in increases of stroke burden since both conditions are major risk factors of stroke. However, not much is known about the prevalence and predictors of stroke among adults with prediabetes and diabetes and yet this information is critical for guiding health programs aimed at reducing stroke burden. Therefore, the objectives of this study were to estimate the prevalence and identify predictors of stroke among persons with either prediabetes or diabetes in Florida.

**Methods:**

The 2019 Behavioral Risk Factor Surveillance System (BRFSS) survey data were obtained from the Florida Department of Health and used for the study. Weighted prevalence estimates of stroke and potential predictor variables as well as their 95% confidence intervals were computed for adults with prediabetes and diabetes. A conceptual model of predictors of stroke among adults with prediabetes and diabetes was constructed to guide statistical model building. Two multivariable logistic models were built to investigate predictors of stroke among adults with prediabetes and diabetes.

**Results:**

The prevalence of stroke among respondents with prediabetes and diabetes were 7.8% and 11.2%, respectively. The odds of stroke were significantly (*p* ≤ 0.05) higher among respondents with prediabetes that were ≥ 45 years old (Odds ratio [OR] = 2.82; 95% Confidence Interval [CI] = 0.74, 10.69), had hypertension (OR = 5.86; CI = 2.90, 11.84) and hypercholesterolemia (OR = 3.93; CI = 1.84, 8.40). On the other hand, the odds of stroke among respondents with diabetes were significantly (*p* ≤ 0.05) higher if respondents were non-Hispanic Black (OR = 1.79; CI = 1.01, 3.19), hypertensive (OR = 3.56; CI = 1.87, 6.78) and had depression (OR = 2.02; CI = 1.14, 3.59).

**Conclusions:**

Stroke prevalence in Florida is higher among adults with prediabetes and diabetes than the general population of the state. There is evidence of differences in the importance of predictors of stroke among populations with prediabetes and those with diabetes. These findings are useful for guiding health programs geared towards reducing stroke burden among populations with prediabetes and diabetes.

## Introduction

Stroke occurs when there is lack of blood supply to a part of the brain due to either blockage of a vessel from a blood clot (ischemic stroke) or bleeding from a ruptured vessel (hemorrhagic stroke) [1]. It is the 5th leading cause of death in the US and more than 600,000 people in the country experience first time stroke each year [2, 3]. It is also the leading cause of long-term serious disability and costs $34 billion each year in the US [3].

Both prediabetes and diabetes are major risk factors of stroke. Individuals with diabetes have two times higher risk of experiencing a stroke than those that do not have the condition [4]. Prediabetes, on the other hand, is a modest risk factor for first time stroke but doubles the risk of recurrent stroke [5, 6]. More than 30 million people in the US have diabetes [2]; the prevalence of the condition has doubled over the last 20 years and is projected to double or triple by 2050. Similarly, the prevalence of prediabetes in the US has been increasing. Currently, almost a third of the US adult population (88 million) have prediabetes [7].

There is evidence that about 23–53% of stroke patients have prediabetes, while 14–46% have diabetes [6, 8]. The increasing trends in prediabetes and diabetes prevalence will likely result in higher stroke burden in the future. Given the changes in prediabetes and diabetes landscape, the American Heart Association (AHA) and American Diabetes Association (ADA) have jointly acknowledged the need to better understand the epidemiology of stroke among individuals that have prediabetes and diabetes so as to better guide stroke prevention and control programs [9–11]. However, not much is known about the epidemiology of stroke in these populations because previous studies have mainly focused on investigating the epidemiology of stroke in the general population [12]. A study conducted in Europe investigated risk factors of stroke among individuals with diabetes, but it did not include the US population [13]. To our knowledge, no study has investigated risk factors of stroke among individuals with prediabetes.

Florida is one of the states in the diabetes belt, an area with a higher burden of the condition than the rest of the country [14]. The burden of diabetes has been increasing in Florida as evidenced by the fact that diabetes prevalence has increased from 5.2% in 1995 to 12.6% in 2018 [15]. This increase will likely result in an increase of Florida’s stroke burden. Understanding stroke epidemiology among individuals with prediabetes and diabetes is important for guiding evidence-based health planning and service provision to control stroke burden and improve quality of care for stroke patients. Therefore, the objectives of this study were to estimate the prevalence and identify predictors of stroke among persons with prediabetes and diabetes in Florida.

## Materials & methods

### Study area

This study was conducted in Florida, the most populous state in the southeastern US with a population of approximately 21.2 million. With 20.4% of the population composed of senior citizens (≥65 years old), Florida has the second-highest number of the elderly population in the US [16]. The rest of the population is distributed as follows: 0–19 years old (22.1%), 20–34 years old (19.1%), 35–44 years old (12.1%), 45–54 years old (12.9%), and 55–64 years old (13.4%). Approximately 51% of the population is female. The majority (77.3%) of the population is White, 16.9% is Black, while all other races comprise 5.8% of the population. By ethnicity, Hispanic-Latino comprises 26.3% of the population, while the rest is non-Hispanic [17].

### Data source, study population and variable selection

The 2019 Behavioral Risk Factor Surveillance System (BRFSS) survey data, obtained from the Florida Department of Health (FDOH), were used in this study. The BRFSS is a telephone survey designed to collect data on individual risk behaviors, chronic health conditions, and preventive health practices from non-institutionalized adults 18 years of age and older. The FDOH conducts the BRFSS survey each year with technical and methodological assistance from the Centers for Disease Control and Prevention (CDC). Respondents are contacted using Random Digit Dialing of telephone numbers obtained from a dual-frame survey of landline and cellular telephones. Disproportionate Sampling Strategy (DSS) and Simple Random Selection (SRS) designs are used to select telephone numbers from the landline and cellular telephone survey frames, respectively [18]. Records of two groups of respondents were extracted from the 2019 BRFSS data, respondents with prediabetes and those with diabetes. The above classification is based on the respondents having been told by a doctor that they had prediabetes or diabetes. No distinction was made between type 1 and type 2 diabetes as this information was not captured in the BRFSS survey. Pregnancy diabetes was excluded since it is a temporary condition. Stroke status was determined based on the respondent’s report of having been told by a doctor that they had a stroke. The survey did not gather information on types of strokes (ischemic, hemorrhagic, and transient ischemic attack) and, therefore, no distinction was made regarding the types of strokes.

**Fig. 1:**
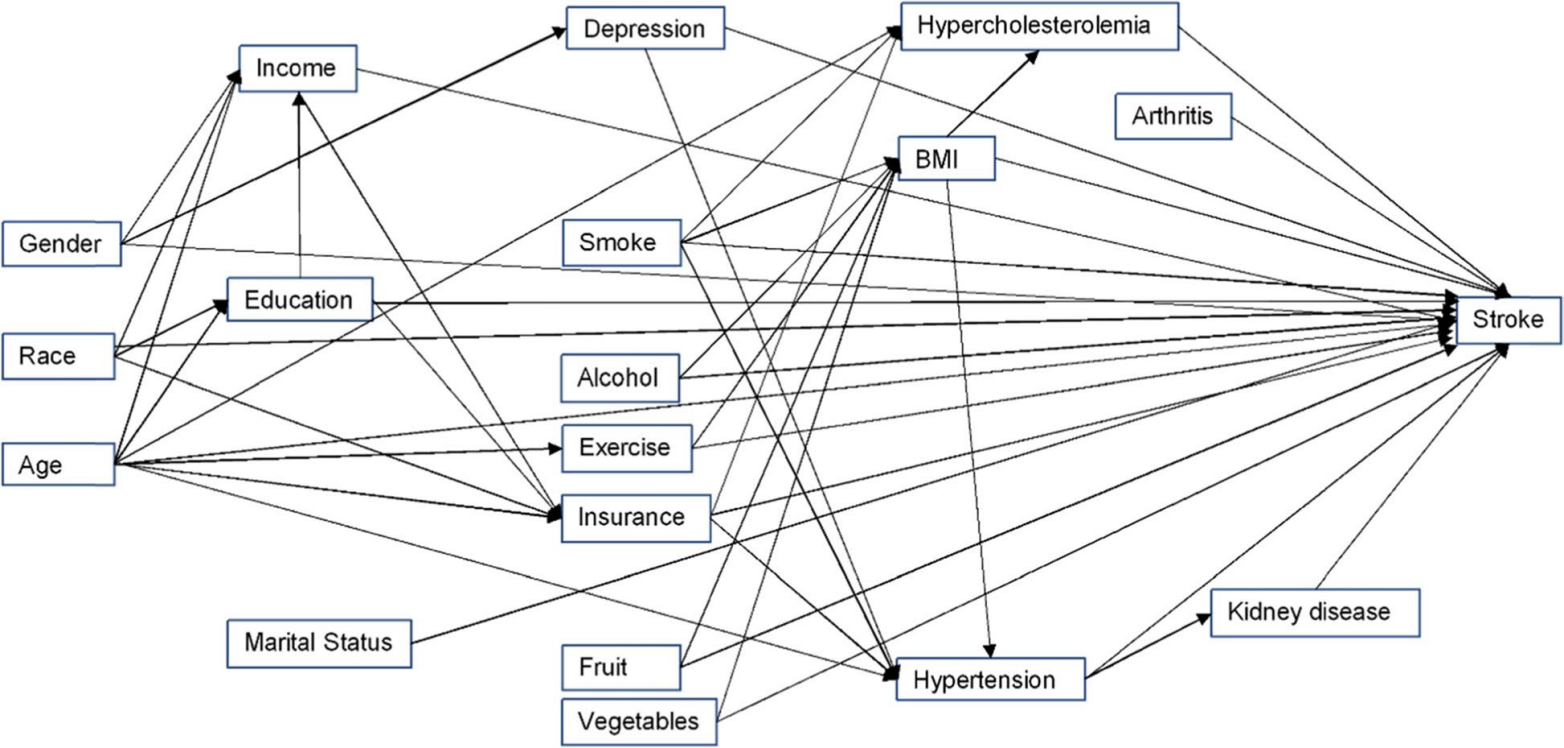
Conceptual model representing predictors of stroke among adults who reported having either prediabetes or diabetes

A conceptual model of predictors of stroke among respondents with prediabetes and diabetes was constructed based on biological knowledge, literature review, and questions asked in the BRFSS survey (Fig. 1). The list of variables considered for investigation as potential predictors or confounders is shown in Table 1. The potential predictors considered for investigation can be broadly classified into sociodemographic factors, risk behaviors, and chronic health conditions. Sociodemographic factors investigated included age, sex, race, marital status, income, education, and health insurance coverage. Risk behaviors investigated were physical activity, Body Mass Index (BMI), fruits and vegetable consumption, if the respondent “ever smoked cigarettes”, and if they had “heavy drinking” habits. Respondents’ physical activities were classified into 4 categories based on the reported duration of physical activity in a week: inactive (no physical activity), insufficiently active (11–149 min/week), active (150–300 min/week), and highly active (> 300 min/week). Based on the BMI score, the BMI status was classified into underweight (< 18.5), normal weight (18.5 to 24.9), overweight (25 to 29.9), and obese (≥30). Respondent’s fruit consumption was classified into 2 categories: consumed ≥1 times/day and < 1 time/day. Similar categorization was used for classifying vegetable consumption. “Ever smoked cigarettes” was determined based on the respondents reports of being a current or former smoker. A respondent was classified as a heavy drinker if an adult male respondent reported having more than 14 drinks per week or an adult female respondent reported having more than 7 drinks per week. Chronic health conditions assessed included hypercholesterolemia (high blood cholesterol), hypertension, arthritis, and kidney disease [18].

**Table 1:** Demographic, health, and lifestyle characteristics among adults who reported having either prediabetes or diabetes

Characteristic	Categories	Prediabetes (n^**a**^ = 1608)		Diabetes (n^**a**^ = 2680)	
		n^**a**^	Weighted % (95% CI^**b**^)	n^**a**^	Weighted % (95% CI^**b**^)
**Age**	65 years or over	785	35.29 (31.00, 39.59)	1663	53.03 (48.88, 57.17)
	45 to 65 years	611	44.66 (39.44, 49.88)	858	38.84 (34.60, 43.08)
	18 to 44 years	212	20.05 (15.30, 24.79)	159	8.13 (6.07, 10.19)
**Sex﻿**	Male	711	45.81 (40.45, 51.16)	1274	52.54 (48.43, 56.65)
	Female	897	54.19 (48.84, 59.55)	1406	47.46 (43.35, 51.57)
**﻿Race**	Other races (non-Hispanic)	99	3.87 (2.30, 5.43)	158	3.98 (2.29, 5.68)
	Hispanic	144	22.18 (16.95, 27.40)	264	22.63 (18.52, 26.73)
	Black (non-Hispanic)	166	14.40 (10.12, 18.68)	383	18.67 (15.28, 22.07)
	White (non-Hispanic)	1199	59.55 (54.26, 64.95)	1875	54.71 (50.85, 58.59)
**Ever smoked cigarettes﻿**	Yes	788	46.37 (41.12, 51.62)	1295	49.01 (44.83, 53.18)
	No	742	53.63 (48.38, 58.88)	1220	50.99 (46.82, 55.17)
**﻿Heavy drinking habit**	Yes	91	6.24 (4.11, 8.38)	65	3.01 (1.77, 4.25)
	No	1401	93.76 (91.62, 95.89)	2411	96.99 (95.75, 98.23)
**﻿Income level**	<$15,000	167	9.84 (6.56, 13.11)	324	14.63 (10.87, 18.37)
	$15,000- < $25,000	263	20.29 (16.14, 24.43)	566	25.75 (21.63, 29.86)
	$25,000- < $35,000	180	13.82 (9.91, 17.73)	299	14.36 (10.12, 18.61)
	$35,000- < $50,000	221	13.86 (10.45, 17.27)	288	13.41 (10.43, 16.39)
	≥$50,000	476	42.19 (36.29, 48.10)	601	31.86 (27.59, 36.13)
**Education**	College	461	21.97 (18.25, 25.70)	640	18.28 (15.78, 20.77)
	Some college	497	35.42 (30.00, 40.83)	764	28.65 (24.84, 32.47)
	High school	502	30.99 (26.27, 35.71)	846	31.09 (27.30, 34.88)
	<High school	147	11.62 (8.19, 15.05)	409	21.98 (18.01, 25.95)
**Depression**	Yes	434	25.19 (21.02, 29.36)	628	20.03 (16.72, 23.34)
	No	1164	74.81 (70.64, 78.98)	2028	79.97 (76.66, 83.28)
**Level of physical activity**	Inactive	502	34.33 (29.12, 39.53)	1070	38.39 (34.50, 42.28)
	Insufficiently active	165	11.58 (7.46, 15.70)	283	16.89 (12.85, 20.92)
	Active	185	16.79 (12.53, 21.05)	265	15.73 (12.04, 19.41)
	Highly active	575	37.30 (31.95, 42.66)	725	29.00 (25.41, 32.59)
**Consume fruit(s)**	≥1 per day	859	65.94 (61.06, 70.81)	1341	60.09 (55.89, 64.28)
	< 1 per day	567	34.06 (29.19, 38.94)	979	39.91 (35.72, 44.11)
**Consume vegetable(s)**	≥1 per day	1120	76.94 (71.48, 82.41)	1773	75.03 (70.96, 79.10)
	< 1 per day	265	23.06 (17.59, 28.52)	471	24.97 (20.90, 29.04)
**Insurance**	Yes	653	80.48 (74.83, 86.13)	831	84.13 (77.84, 90.41)
	No	150	19.52 (13.87, 25.17)	163	15.87 (9.59, 22.16)
**Hypercholesterolemia**	Yes	848	48.37 (42.97, 53.76)	1569	59.68 (55.41, 63.96)
	No	676	51.63 (46.24, 57.03)	979	40.32 (36.04, 44.59)
**Hypertension**	Yes	974	53.73 (48.34, 59.11)	2009	72.06 (68.02, 76.09)
	No	632	46.27 (40.89, 51.66)	664	27.94 (23.91, 31.98)
**Body Mass Index (BMI) (kg/m**^**2**^**)**	Underweight	25	2.14 (0.31, 3.97)	14	0.27 (0.03, 0.51)
	Normal (18.5–24.9)	252	17.41 (12.93, 21.90)	381	17.16 (13.44, 20.89)
	Overweight (25–29.9)	518	31.99 (27.34, 36.64)	833	35.99 (31.70, 40.28)
	Obese (≥30)	675	48.46 (42.88, 54.04)	1200	46.58 (42.27, 50.88)
**Marital Status**	Never married	209	14.54 (10.82, 18.26)	268	11.56 (9.17, 13.95)
	Separated/divorced/widowed	634	30.47 (25.57, 35.38)	1185	35.70 (31.81, 39.59)
	Married	765	54.95 (49.74, 60.24)	1227	52.74 (48.58, 56.90)
**﻿Arthritis**	Yes	787	38.73 (34.05, 43.42)	1373	49.02 (44.87, 53.17)
	No	812	61.27 (56.58, 65.95)	1292	50.98 (46.83, 55.13)
**﻿Chronic kidney disease**	Yes	87	5.79 (2.80, 8.78)	343	12.07 (9.37, 14.77)
	No	1513	94.21 (91.22, 97.20)	2314	87.93 (85.23, 90.63)
**Stroke﻿**	Yes	142	7.77 (5.25, 10.28)	361	11.16 (8.64, 13.69)
	No	1462	92.23 (89.72, 94.75)	2300	88.84 (86.32, 91.36)

^a^*CI* Confidence intervals

^b^*n* Unweighted sample size

### Data preparation and descriptive analyses

Race/ethnicity was re-coded into four categories (Non-Hispanic White, Non-Hispanic Black, Hispanic, and Other). The “Other” category included non-Hispanic Asian, American Indian/Alaskan Native, or all other races not listed above. Age was categorized into 18–44 years, 45–64 years, and 65 years or older and marital status was classified into married; divorced, widowed, or separated; and never married.

Since the BRFSS data were collected using a complex survey design, a weight variable (created by the US Centers for Disease Control and Prevention [CDC]) was used in all analyses to ensure that the estimates are generalizable to all Florida adults. Descriptive analyses were conducted in SAS using SURVEY codes [19] specifying strata variable (_STSTR), cluster variable (_PSU) and a sampling weight variable (_LLCPWT). Weighted percentage and 95% confidence intervals (95% CI) were calculated for all categorical variables.

### Predictors of stroke among population with prediabetes and diabetes

Two multivariable logistic regression models were built to investigate predictors of stroke among respondents with prediabetes (model 1) and those with diabetes (model 2). The process of model building was similar in both models. The model building process involved first assessing univariable associations between each potential predictor and the outcome (stroke among either prediabetes [for model 1] or diabetes [for model 2] respondents) using a liberal *p*-value of ≤0.20. To avoid multicollinearity in subsequent multivariable models to be built, two-way Spearman rank correlation analyses were performed on all variables that showed significant association (based on relaxed *p* ≤ 0.20) in the univariable analyses. Only one of a pair of highly correlated variables (r > 0.7) was considered for assessment in the subsequent multivariable models. The choice of which of a pair of highly correlated potential predictors to be assessed in the multivariable models was based on biological and statistical considerations. Manual backward elimination procedures were then used to fit the final weighted multivariable models setting the *p*-values for removal at ≤0.05. Confounders were assessed using changes in regression coefficients of variables in the models when the models were run with and without suspected confounders. If removal of a suspected confounding variable resulted in a change of 20% or more of any of the other variables in the models, then the variable was retained in the models as a confounder regardless of its statistical significance. Variables that had either significant association with the outcome or confounding effect were retained in the final main-effects models. Age was forced in the models due to a priori belief that it was a confounder. Biologically meaningful two-way interaction terms of the variables were then assessed with the aim of keeping significant ones in the final models. Odds ratios and 95% confidence intervals (95% CI) were computed for all predictors in the final main-effects models. Goodness-of-fit of the models were assessed using the Hosmer-Lemeshow test [20]. All statistical analyses were performed in SAS 9.4 [19].

## Results

### Prevalence estimates

The study included a total of 16,959 survey respondents, of whom 9.5% (1608) and 15.8% (2680) had been told by a doctor that they had prediabetes and diabetes, respectively. Table 1 shows the demographic, health, and lifestyle characteristics of respondents that had prediabetes and diabetes. Most of the respondents with prediabetes were between 45 and 65 years old (44.7%), female (54.2%), non-Hispanic White (59.6%), and married (55.0%). The majority of them had an annual income of ≥$50,000 (42.2%), some college education (35.4%), and health care coverage (80.5%). Regarding risk behaviors, respondents were mostly non-smokers (53.6%), did not have depression (74.8%), had heavy drinking habits (93.8%), and reported consuming fruits (66.0%) and vegetables (77%) at least once a day. However, a lower percentage reported being highly physically active (37.3%). Almost half of the respondents with prediabetes reported being obese (48.5%), having hypercholesterolemia (49.4%) and hypertension (53.7%). Thirty-eight percent of the respondents had arthritis, while a few had chronic kidney diseases (5.8%).

Respondents with diabetes were predominantly ≥65 years old (53%), male (52.5%), non-Hispanic White (54.7%), and married (52.7%). Thirty-one percent of the respondents had high school education and 31.9% reported earning ≥$50,000 annually. Unlike respondents with prediabetes, most of those with diabetes had hypercholesterolemia (59.7%), hypertension (72.3%), and were physically inactive (38.4%). Similar to the respondents with prediabetes, the majority of those with diabetes were obese (46.6%), non-smokers (51.0%), did not have heavy drinking habits (97.0%), had healthcare coverage (84.1%), and consumed fruits (60.1%) and vegetables (75.0%) at least once a day. The percentage of stroke among respondents that had prediabetes and diabetes was 7.8 and 11.2%, respectively.

### Univariable associations

Table 2 shows the univariable (unadjusted) associations of each of the predictors with stroke among respondents with prediabetes and diabetes in Florida. The following variables had potentially significant (*p* ≤ 0.20) simple/univariable associations with stroke among individuals with prediabetes: age, sex, income, depression, physical activity, vegetable consumption, hypercholesterolemia, hypertension, BMI, arthritis, and chronic kidney disease. All the above variables, except age, sex, and vegetable consumption, also had potentially significant (*p* ≤ 0.20) associations with stroke among individuals with diabetes. Additionally, race and ever smoking cigarettes were significantly associated with stroke among individuals with diabetes but not among those with prediabetes.

**Table 2:** Univariable associations of potential predictors of stroke among adults with prediabetes and diabetes in Florida

Characteristic	Categories	Respondents with prediabetes		Respondents with diabetes	
		Unadjusted Odds Ratio (95% CI^**a**^)	***p***-value	Unadjusted Odds Ratio (95% CI^**a**^)	***p***-value
**Age**	65 years or over	5.57 (1.52, 20.41)	< 0.001	1.56 (0.55, 4.40)	0.418
	45 to 65 years	12.61 (3.68, 43.24)		1.99 (0.66, 5.97)	
	18 to 44 years	Reference		Reference	
**Sex**	Male	1.56 (0.79, 3.10)	0.200	1.32 (0.81, 2.14)	0.266
	Female	Reference		Reference	
**Race﻿**	Other races (non-Hispanic)	2.08 (0.69, 6.24)		0.49 (0.24, 1.04)	0.027﻿
	Hispanic	0.66 (0.21, 2.08)	0.320	1.17 (0.54, 2.56)	
	Black (non-Hispanic)	0.50 (0.12, 2.10)		1.87 (1.04, 3.38)	
	White (non-Hispanic)	Reference		Reference	
**Ever smoked cigarettes﻿**	Yes	1.42 (0.69, 2.91)	0.340	1.55 (0.92, 2.63)	0.103
	No	Reference		Reference	
**Heavy drinking habit﻿**	Yes	0.56 (0.13, 2.38)	0.430	0.75 (0.19, 3.00)	0.687
	No	Reference		Reference	
**Income level﻿**	<$15,000	0.78 (0.28, 2.21)		2.26 (0.91, 5.62)	﻿0.038
	$15,000- < $25,000	1.01 (0.37, 2.76)		1.98 (1.01, 3.89)	
	$25,000- < $35,000	3.64 (1.22, 10.86)	0.069	2.05 (0.72, 5.87)	
	$35,000- < $50,000	1.87 (0.64, 5.49)		0.78 (0.35, 1.77)	
	≥$50,000	Reference		Reference	
**Education**	College	0.41 (0.12, 1.34)		0.71 (0.31, 1.60)	
	Some college	0.39 (0.14, 1.12)	0.260	0.65 (0.30, 1.42)	0.751
	High school	0.38 (0.14, 1.04)		0.68 (0.31, 1.49)	
	<High school	Reference		Reference	
**Depression**	Yes	2.22 (1.08, 4.59)	0.030	2.07 (1.18, 3.63)	0.012
	No	Reference		Reference	
**Level of physical activity**	Inactive	1.49 (0.64, 3.45)		1.22 (0.67, 2.24)	﻿0.183
	Insufficiently active	0.37 (0.10, 1.33)	0.178	0.54 (0.24, 1.21)	
	Active	0.98 (0.30, 3.23)		1.09 (0.38, 3.13)	
	Highly active	Reference		Reference	
**Consume fruit(s)**	≥1 per day	0.71 (0.34, 1.50)	0.366	1.21 (0.71, 2.05)	0.481
	< 1 per day	Reference		Reference	
**Consume vegetable(s)**	≥1 per day	1.94 (0.81, 4.62)	0.136	0.85 (0.45, 1.62)	0.622
	< 1 per day	Reference		Reference	
**Insurance**	Yes	0.70 (0.29, 1.67)	0.417	1.31 (0.37, 4.67)	0.675
	No	Reference		Reference	
**Hypercholesterolemia**	Yes	5.40 (2.57, 11.35)	< 0.001	1.82 (1.04, 3.16)	0.035
	No	Reference		Reference	
**Hypertension**	Yes	7.33 (3.64, 14.78)	< 0.001	3.78 (2.15, 6.67)	< 0.001
	No	Reference		Reference	
**Body Mass Index (BMI) (kg/m**^**2**^**)**	Under weight (< 18.5)	0.73 (0.15, 3.58)		8.08 (1.27, 51.40)	0.072
	Normal (18.5–24.9)	Reference	0.181	Reference	
	Overweight (25–29.9)	2.02 (0.70, 5.83)		0.75 (0.37, 1.53)	
	Obese (≥30)	0.94 (0.33, 2.73)		0.98 (0.48, 2.02)	
**Marital Status**	Never married	0.87 (0.28, 2.72)	0.379	1.03 (0.46, 2.31)	0.954
	Separated/divorced/widowed	1.62 (0.77, 3.42)		1.09 (0.63, 1.89)	
	Married	Reference		Reference	
**﻿Arthritis**	Yes	2.38 (1.17, 4.83)	0.017	1.58 (0.95, 2.64)	0.078
	No	Reference		Reference	
**﻿Chronic kidney disease**	Yes	3.10 (0.89, 10.75)	0.075	2.36 (1.14, 4.87)	0.020
	No	Reference		Reference	

^a^*CI* Confidence Intervals

### Predictors of stroke among respondents with prediabetes and diabetes

The results of the final multivariable logistic regression models are presented in Table 3. Significant predictors of the odds of stroke among respondents with prediabetes were age, hypertension, and hypercholesterolemia. The odds of stroke among individuals 45–65 years old with prediabetes were 2.82 times [95% Confidence Interval (CI): 0.74, 10.69] higher than among those who were 18–44 years old while that among individuals ≥65 years with prediabetes were even higher [Odds Ratio (OR) = 4.90; 95% CI: 1.38, 17.45]. The odds of stroke among individuals with prediabetes that also had hypertension were also higher (OR = 5.86; 95% CI: 2.90, 11.84) than those among individuals that had prediabetes but not hypertension. Similarly, the odds of stroke among individuals that had prediabetes as well as hypercholesterolemia were 3.93 (95% CI: 1.84, 8.40) times higher than among individuals that had prediabetes but not hypercholesterolemia.

**Table 3:** Final multivariable logistic regression models showing predictors of stroke among adults with prediabetes and diabetes

Characteristic	Categories	Respondents with prediabetes (model 1)		Respondents with diabetes (model 2)	
		Adjusted Odds Ratio (95% CI^**a**^)	***p***-value	Adjusted Odds Ratio (95% CI^**a**^)	***p***-value
**Age**	65 years or over	4.90 (1.38, 17.45)	0.032	1.68 (0.56, 5.05)	0.352
	45 to 65 years	2.82 (0.74, 10.69)		2.19 (0.69, 6.97)	
	18 to 44 years	Reference		Reference	
**Hypertension﻿**	Yes	5.86 (2.90, 11.84)	< 0.001	3.56 (1.87, 6.78)	< 0.001
	No	Reference		Reference	
**Hypercholesterolemia﻿**	Yes	3.93 (1.84, 8.40)	< 0.001		
	No	Reference			
**﻿Race**	Hispanic			1.23 (0.56, 2.70)	0.025
	Black (non-Hispanic)			1.79 (1.01, 3.19)	
	Other races (non-Hispanic)			0.42 (0.18, 0.96)	
	White (non-Hispanic)			Reference	
**﻿Depression**	Yes			2.02 (1.14, 3.59)	0.016
	No			Reference	

^a^*CI* Confidence Intervals

None of the significant predictors of stroke among individuals with prediabetes, except hypertension, was significantly associated with stroke among individuals with diabetes. The odds of stroke among individuals with diabetes that also had hypertension were 3.56 times (95% CI: 1.87, 6.78) higher than the odds of stroke among individuals that had diabetes but normal blood pressure. In addition, non-Hispanic Black (OR = 1.79, 95%CI: 1.01, 3.19) and depressed (OR = 2.02, 95% CI: 1.14, 3.59) individuals with diabetes had higher odds of stroke compared to non-Hispanic White and non-depressed individuals with diabetes, respectively. Although age was not a significant predictor of stroke among individuals with diabetes, it was retained in the final model because of a priori knowledge that it is a confounder. It is worth noting that non-Hispanic individuals of other races (OR = 0.42, 95% CI: 0.18, 0.96) had significantly lower odds of stroke than non-Hispanic White among individuals with diabetes.

## Discussion

This study investigated the prevalence and predictors of stroke among adults who reported having either prediabetes or diabetes in Florida. The percentages of stroke among adults with prediabetes (7.8%) and diabetes (11.2%) were higher than among the general population of Florida (3.6%) in 2019 [21]. No previous studies have investigated predictors of stroke among adults with prediabetes and diabetes and yet this information is critical for guiding health programs aimed at reducing stroke burden in Florida.

The identification of hypertension as a common predictor of stroke among adults with prediabetes and diabetes in this study is consistent with the findings from previous studies [22–24]. According to a report by the AHA, hypertension increases risk of stroke by weakening arteries and weakened arteries are more likely to burst or clog resulting in hemorrhagic or ischemic stroke, respectively [24]. Evidence suggests that persons with prediabetes and diabetes have damaged blood vessels and compromised functionalities of heart and kidney due to higher than normal blood glucose levels [25, 26]. Compromised kidney functions increase blood volume and again decrease the stretching capacity of blood vessels [25, 26]. As a result, adults with prediabetes and diabetes are more likely to experience stroke if they also have hypertension.

Previous studies reported age as a non-modifiable risk factor of stroke among both males and females [27–29]. Similar to these findings, this study identified higher odds of stroke among individuals ≥45 years old who had prediabetes [27–29]. A study by Bushnell et al. reported that the risk of stroke doubles every 10 years after age 55 [30]. The possible mechanism underlying the effect of age is that arteries naturally became narrower and harder with increasing age due to the change mediated by endothelial dysfunction and impaired cerebral autoregulation [28]. Moreover, certain stroke risk factors such as diabetes, hypertension, atrial fibrillation, and coronary and peripheral artery diseases steadily increase with age [28]. However, evidence also suggests that adolescents and younger people aged 15–49 years also have a high risk of stroke due to obesity and high blood pressure [31]. In contrast to the previous findings, age was not significantly associated with stroke risks among persons with diabetes in this study [27–29]. The reason for this remains unclear. However, two or more comorbidities are quite common among older individuals with diabetes, which could potentially interact with conventional cardiovascular risk factors (i.e., age) to increase the risk of stroke [28].

Similar to the findings of this study, several studies suggested that high blood cholesterol levels increased risk of stroke [32, 33]. Higher odds of stroke among individuals with both prediabetes and hypercholesterolemia could be explained by the changes of lipid metabolism among these populations. Adults with prediabetes have distinctive form of dyslipidemia characterized by low levels of High Density Lipoprotein (HDL)-cholesterol and moderately elevated levels of Triglyceride (TG)-rich lipoprotein [34]. Dysmetabolism of TG-rich lipoprotein increases the level of smaller and denser Low-Density Lipoprotein (LDL) particles. Overall, non-HDL cholesterol levels, including TG and LDL, almost always increase among adults with prediabetes. Moreover, increased non-HDL cholesterol levels due to lipid dysmetabolism and weakened blood vessels due to hyperglycemia among adults with prediabetes increase the risk of atherosclerosis and ischemic stroke [35]. Surprisingly, hypercholesterolemia was not a significant predictor of stroke among adults with diabetes in this study. This is possibly due to the fact that anti-diabetic medications such as sulfonylurea and insulin can control hypercholesterolemia and, to some extent, reduce the risk of developing stroke [36, 37].

Risks of stroke among populations with diabetes vary by race. The higher odds of stroke among non-Hispanic Black compared to non-Hispanic White, identified in this study, is consistent with reports from previous studies [38–41]. There is evidence that non-Hispanic Black populations have high risks of stroke because they are more likely to have hypertension and diabetes [39–41]. However, another study by Heyman et al. suggested that even after adjusting for hypertension and diabetes, non-Hispanic Black individuals had consistently higher risk of stroke than non-Hispanic White [38, 42]. Studies have reported that only half of the excess risk of stroke among non-Hispanic Black could be attributed to traditional risk factors (such as poor diet, obesity, and high salt diet), implying that genetic and biological factors might have potential roles in stroke disparities among non-Hispanic Black population [43–45]. Additionally, non-Hispanic Black adults with diabetes often do not have access to healthcare due to low socioeconomic conditions and tend to have uncontrolled diabetes [46]. The presence of inherent excess risk of stroke and uncontrolled diabetes may be responsible for higher odds of stroke among non-Hispanic Black individuals with diabetes compared to non-Hispanic White individuals with diabetes. Although Hispanic individuals had seemingly higher odds of stroke than non-Hispanic White individuals, this association was not statistically significant. A study by Rodriguez et al. also reported that age-adjusted prevalence of stroke among Hispanic individuals ≥18 years were similar to stroke prevalence among their non-Hispanic White counterparts [47]. However, this relationship may vary by geographic region as several studies, conducted in other US states, reported a significantly higher risk of stroke among Hispanic individuals compared to their non-Hispanic White counterparts. Similarly, the risk of stroke among non-Hispanic other races, including Asian and American Indian/Alaskan Native, also vary by geographic location [48]. This study identified significantly lower risk of stroke among non-Hispanic other races than non-Hispanic White individuals in Florida, while studies in other US states reported the opposite [49–51]. However, it is worth pointing out that non-Hispanic other races represent a small portion of the Florida population. Overall, reasons for identified disparities in stroke risks among minority populations could be genetic and higher prevalence of traditional risk factors such as diabetes, hypertension, low socioeconomic status, and healthcare system challenges [52]. Surprisingly, race was not a significant predictor of stroke among adults with prediabetes. The reason for this is not apparent but may be due to the fact that other factors such as age, hypertension, hypercholesterolemia are more important predictors of stroke among these populations.

Based on the findings from this study, adults that had both diabetes and depression had two times higher odds of stroke compared to those that had diabetes but no depression and this finding is consistent with those from a meta-analysis of 17 epidemiological prospective studies showing significant positive associations between depression and stroke even after adjusting for diabetes, hypertension, and other risk factors [53]. Individuals experiencing depression tend to have unhealthy lifestyles, get less exercise, often times do smoke, and are more likely to miss prescribed medication [54]. Other possible mechanisms linking depression to stroke could be inflammation, atherosclerosis, lesions in cerebral white matter, cardiac arrhythmia, and increased platelet activity [53, 54].

Since estimates obtained from the BRFSS data are representative of the adult population of Florida, they are generalizable to Florida adults. Thus, the findings from this study are useful for guiding health planning for the state of Florida. However, Florida population could also be representative of similar populations in other high- and middle-income countries with similar socioeconomic structure and quality of life. Moreover, identified predictors of stroke in this study such as hypertension and hypercholesterolemia are common comorbidities among populations all over the world. Therefore, the findings of this study could provide guidance to conduct similar studies among populations of other high- or middle-income countries. However, it is worth noting that differences in healthcare systems, environment, and inherent biological factors among populations of different races and ethnicities in different countries could result in the identification of different sets of predictors of stroke.

### Strengths and limitations

To our knowledge, this is the first study that estimated the prevalence and investigated predictors of stroke among populations with prediabetes. The findings are critical for reducing stroke burden considering the fact that populations with prediabetes represent 1/3 of the US adult population. This is also the first study investigating predictors of stroke among populations with diabetes in Florida. Identifying populations that have prediabetes or diabetes with a high risk of stroke will help enhance evidence-based programs targeting those populations in Florida. These findings are important as the Florida Department of Health seeks to implement the new Paul Coverdell National Acute Stroke Program which aims to improve the quality of care for stroke patients. A strength of the BRFSS data is that it is based on statistical survey sampling methodology and therefore provide representative estimates that are generalizable to the population. However, this study is not without limitations. First, since the BRFSS was based on self-reported responses, prevalence estimates calculated from this survey could be affected by reporting or recall biases. For instance, it is possible that people who had not suffered a severely disabling stroke might forget to mention having a stroke (recall bias). However, these will bias the estimates of associations towards the null, implying that the true associations are even stronger than the results shown in this study. Additionally, since the study used secondary data, there was no way to cross-check medical records with self-reported responses in the BRFSS survey. However, studies have shown that BRFSS data are accurate even after considering reporting bias [55, 56]. Secondly, rates of detection of prediabetes or diabetes might be higher among individuals of higher socioeconomic status than those in low status. Therefore, more affluent individuals might be overrepresented among those reporting prediabetes and diabetes [57]. In addition, the BRFSS did not collect data on: (a) the duration of prediabetes and diabetes, (b) medications for management of stroke risk factors (i.e., hypertension and hypercholesterolemia), and (c) effectiveness of control of blood sugar, blood pressure, and cholesterol among populations with prediabetes and diabetes. Therefore, the impacts of these on the risk of stroke could not be assessed. Finally, the BRFSS survey did not gather information on types of stroke and hence stroke risks could not be investigated based on types of stroke. These limitations notwithstanding, the findings of this study provide useful information to guide health planning and programs aimed at reducing stroke burden in Florida.

## Conclusions

This study has shown evidence of higher prevalence of stroke among populations with prediabetes and diabetes than the general population in Florida. Study findings also provide some evidence that there may be differences in the importance of predictors of stroke among adults with prediabetes and those with diabetes. Regular checkups and controlling blood pressure and cholesterol levels among adults with prediabetes could help reduce stroke risks. On the other hand, non-Hispanic Black and Hispanic adults with diabetes are of specific concern as they have higher odds of stroke and represent almost half of the Florida population that have diabetes. Study findings will be useful in guiding health equity programs geared towards reducing stroke burden among populations with prediabetes and diabetes in Florida. Future studies could use similar methods to estimate stroke burden and identify common predictors of stroke among the overall US population as well as populations of high-, middle-, or low-income countries that have prediabetes and diabetes.

## Data Availability

The datasets generated and/or analyzed during the current study are not publicly available because they belong to a third party and the authors do not have legal authority to share the data but are available from the Florida Department of Health at 850–245-4793 or BRFSS@flhealth.gov on reasonable request.

## Abbreviations

AHA: American Heart Association
ADA: American Diabetes Association
US: United States
BRFSS: Behavioral Risk Factor Surveillance System
FDOH: Florida Department of Health
CDC: Centers for Disease Control and Prevention
BMI: Body Mass Index
OR: Odds Ratio
CI: Confidence Interval
HDL: High Density Lipoprotein
LDL: Low Density Lipoprotein
TG: Triglyceride
